# The epidemiology of dengue outbreaks in 2016 and 2017 in Ouagadougou, Burkina Faso

**DOI:** 10.1016/j.heliyon.2020.e04389

**Published:** 2020-07-14

**Authors:** Justin Im, Ruchita Balasubramanian, Moussa Ouedraogo, Lady Rosny Wandji Nana, Ondari D. Mogeni, Hyon Jin Jeon, Tayma van Pomeren, Andrea Haselbeck, Jacqueline Kyungah Lim, Kristi Prifti, Stephen Baker, Christian G. Meyer, Jerome H. Kim, John D. Clemens, Florian Marks, Abdramane Bassiahi Soura

**Affiliations:** a International Vaccine Institute, Seoul, Republic of Korea; b Princeton University, Princeton, NJ, USA; c Laboratorie d'Analyses Medicales, Centre Médical avec Antenne chirurgicale Protestant Schiphra, Ouagadougou, Burkina Faso; d Institut Supérieur des Sciences de la Population, Université de Ouagadougou, Ouagadougou, Burkina Faso; e The Department of Medicine, University of Cambridge, Cambridge, United Kingdom; f Duy Tan University, Da Nang, Viet Nam; g Institute for Tropical Medicine, Eberhard Karls University, Tübingen, Germany; h International Centre for Diarrheal Disease Research, Bangladesh, Dhaka, Bangladesh; i University of California, Fielding School of Public Health, Los Angeles, USA

**Keywords:** Viruses, Viral disease, Epidemiology, Public health, Infectious disease, Dengue, Outbreak, Burkina Faso, Serotypes

## Abstract

**Background:**

Dengue is prevalent in as many as 128 countries with more than 100 million clinical episodes reported annually and four billion people estimated to be at risk. While dengue fever is systematically diagnosed in large parts of Asia and South America, the disease burden in Africa is less well investigated. This report describes two consecutive dengue outbreaks in Ouagadougou, Burkina Faso in 2016 and 2017.

**Methods:**

Blood samples of febrile patients received at Schiphra laboratory in Ouagadougou, Burkina Faso, were screened for dengue infection using SD Bioline Dengue Duo rapid diagnostic test kits (Standard Diagnostics, Suwon, Republic of Korea).

**Results:**

A total of 1,397 and 1,882 cases were reported by a single laboratory in 2016 and 2017, respectively. Most cases were at least 15 years of age and the results corroborated reports from WHO indicating the circulation of three dengue virus serotypes in Burkina Faso.

**Conclusion:**

This study complements data from other, simultaneously conducted surveillance efforts, and indicates that the dengue disease burden might be underestimated in sub-Saharan African nations. Dengue surveillance should be enhanced in African settings to determine the burden more accurately, and accelerated efforts towards a dengue vaccine should be put in place.

## 1 Introduction

Dengue infection is the most common mosquito-transmitted (genus *Aedes*) viral disease with more than 100 million annual symptomatic episodes being reported from as many as 128 countries; approximately four billion people are estimated to be at risk [1, 2, 3]. Yet, given a high asymptomatic infection rate, the actual burden of dengue is potentially significantly higher, with estimates ranging to up to four times more [4]. The highest numbers of dengue cases are reported in South America and Asia [2, 3]. Being an urban disease, it principally arises in densely populated areas, aided by *Aedes aegypti* breeding preferences.

While dengue is in most settings solely diagnosed based on the clinical picture, the disease can be confirmed by a variety of diagnostic methods including the detection of anti-dengue virus (DENV) antibodies, non-structural protein 1 (NS1) antigen or DENV-specific nucleic acid detection. Yet, confirmatory testing, especially in resource-limited settings, is often not executed.

This is particularly the case in many African settings, and even though dengue cases have been reported in 34 African countries, the epidemiology of the disease in Africa is largely undescribed as a consequence of limited disease surveillance, low awareness, and poor diagnostic facilities [5]. Only 20 laboratory-confirmed outbreaks were reported from 15 sub-Saharan African countries in the period between 1960 and 2010; however, an additional 300,000 cases were reported from epidemics in the Seychelles, Réunion, Djibouti, the Comoros, and Cape Verde [6]. More recently, following improved diagnostics and increased public awareness, there have been dengue cases and associated deaths reported in Kenya, Somalia, Tanzania, Mozambique, and Sudan [7].

From August to November 2016, the World Health Organization (WHO) reported a dengue outbreak in Burkina Faso. From 1,266 suspected cases, there were 1,061 probable cases and 15 deaths [8]. Blood samples from 61 probable cases were dispatched to the Institut Pasteur in Dakar, Senegal, for confirmation; 29 (47.5%) were DENV-2 positive by qRT-PCR [8]. In April 2017, the WHO reported an outbreak in Ivory Coast [9] which then spread to Burkina Faso on August 6^th^, 2016. This outbreak consisted of 9,029 suspected cases, 63.9% (5,773/9,029) of these were positive by rapid diagnostic test (RDT) and 18 deaths were reported [10]. Two hundred and forty-one blood samples were subjected to DENV-PCR at the Centre Muraz (the national viral hemorrhagic fever reference laboratory, Bobo-Dioulasso) of which 58.5% (141/241) were positive by PCR. Subtyping of 72 PCR-positive samples revealed 58, 12, and 2 samples positive for DENV-2, -3, and -1 serotypes, respectively [10]. At the same time, a surveillance study conducted by Lim et al. in adjacent areas in Ouagadougou from December 2014 to February 2017 encompassing the WHO reported outbreak of August–November 2016 reported 2,929 fever cases during both outbreak and non-outbreak periods; 25.3% (740/2,929) were dengue-positive [11]. Seventy-three percent (540/740) of the dengue-positive cases were laboratory-confirmed and 27.0% (200/740) were classified as probable dengue; among these 42.8% (317/740) were confirmed by RT-PCR and the remainder by paired ELISA [11]. During the outbreak period, DENV-2 predominated [70% (181/258) in samples confirmed by RT-PCR], during the non-outbreak period, DENV-3 was mostly observed [65% (28/43) in samples confirmed by RT-PCR] with a few DENV-1 [12% (5/43) in samples confirmed by RT-PCR] [11].

## 2 Methods

**Figure 1:**
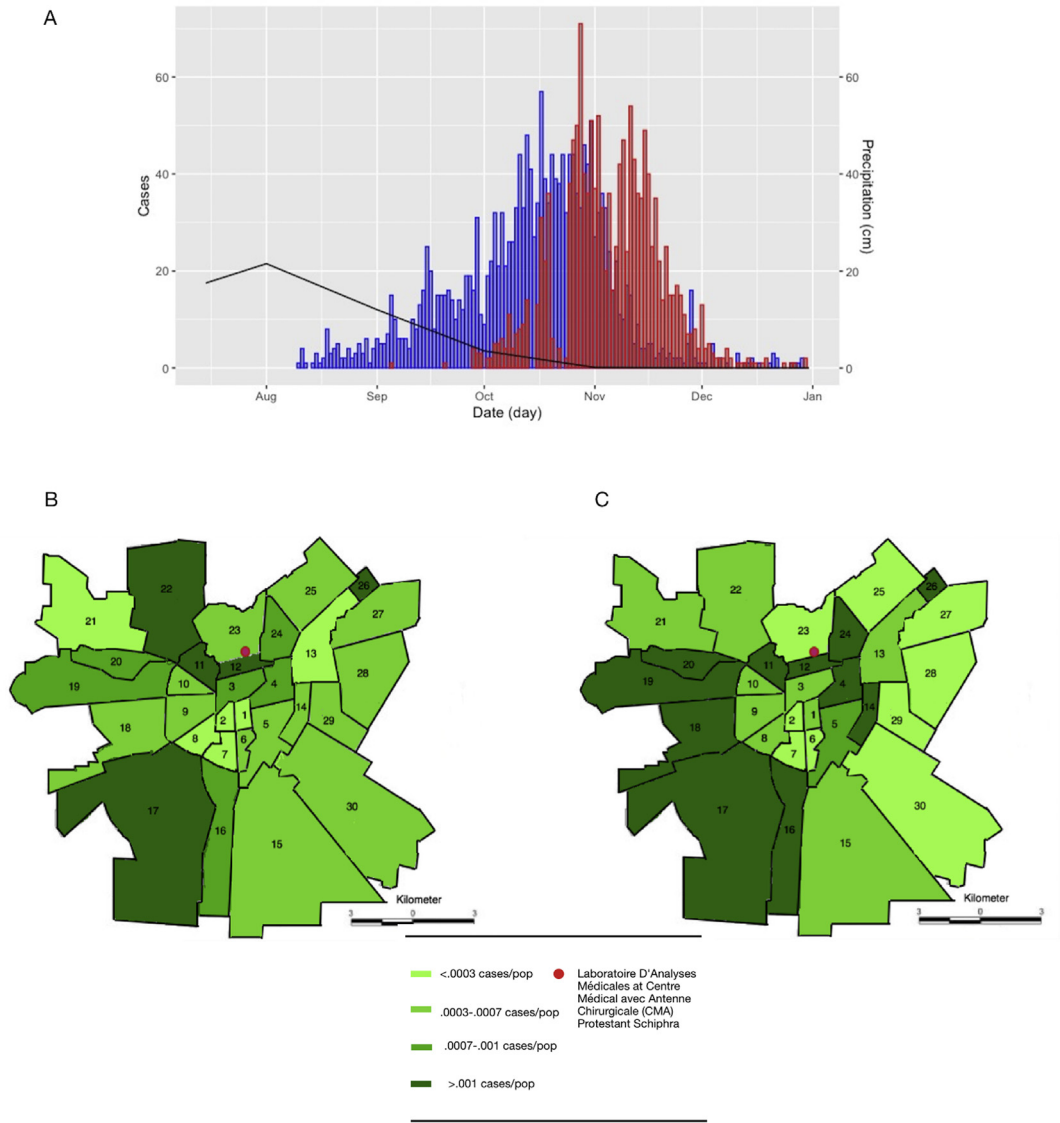
**Number and geographic distribution of dengue cases in Ouagadougou (2016–2017).** (A) Epidemic curve for the 2016 (red) and 2017 (blue) Dengue infection outbreak. The average monthly precipitation in cm is plotted in black [14]. (B/C) Geographic distribution of the number of cases in each sector stratified by population. The majority of cases were reported from the west/south-west of Ouagadougou in both outbreaks. The red dot depicts the location of the CMA Schiphra laboratory.

In collaboration with the Laboratoire D'Analyses Médicales at Centre Médical avec Antenne Chirurgicale (CMA) Protestant Schiphra in Ouagadougou, Burkina Faso, we investigated the dengue outbreaks (Figure 1) using samples collected at CMA. The suspected dengue cases originated from either Schiphra hospital or from surrounding healthcare facilities; all diagnostic samples were received in the CMA laboratory. Samples used for this investigation originated from routine diagnosis of patients. Blood samples received at the CMA from febrile patients were screened for dengue infection using the SD Bioline Dengue Duo RDT which detects the dengue virus NS1 antigen and dengue IgM/IgG antibodies (Standard Diagnostics, Suwon, Republic of Korea). This test was found to have an overall sensitivity and specificity of 93.9% and 92.0%, respectively, in a cohort study conducted in Singapore [12]. A more recent study conducted in Brazil contrasted those results by attributing the test satisfactory levels of specificity (88%–96%), yet, lower overall sensitivity of 46.8% [13]; this test was also used in patient screening in the surveillance study conducted by Lim et al. [11] in other parts of Ouagadougou. *Plasmodium falciparum* malaria was detected by microscopy or using the SD Bioline Malaria Pf RDT (Standard Diagnostics, Suwon, Republic of Korea). Information on date of birth, sex, address, and date of disease onset was collected as standard practice. Data were extracted without personal identifiers; therefore, this study was deemed to be exempt from ethical review by the Institutional Review Board at the International Vaccine Institute. Statistically, categorical variables were compared and tested using the Pearson's Chi-square test with Yates' continuity correction. A map of Ouagadougou delineating administrative sectors was created using Adobe Illustrator CC Software (Adobe Systems, San Jose, CA, USA) to show the location number of the RDT-confirmed dengue cases per sector during the 2016 and 2017 outbreaks (Figure 1). All data analyses were performed in Microsoft Excel (Microsoft, Redmond, WA, USA) and RStudio (RStudio, Boston, MA, USA).

## 3 Results

Laboratory logbooks were retrospectively screened to identify probable and confirmed dengue cases; 4,939 and 7,498 records were screened from 2016 and 2017, respectively. Cases referred to CMA from surrounding healthcare facilities accounted for 65% (8,084/12,437) of assessed cases. The remaining cases originated from Schiphra hospital. From September 5 to December 31, 2016, 28.3% (1,397/4,939) of patients presenting with fever to Schiphra hospital tested positive for dengue virus. This first dengue outbreak peaked on October 28 with 72 identified cases (Figure 1A). An additional 432 cases had been reported by the time the outbreak had subsided in early December. The largest proportion of cases was identified in patients aged ≥15 years (75.8%; 1,059/1,397), followed by children aged <2 years (13.8%; 193/1,397). Among the confirmed dengue cases, 49.7% (694/1,397) were male and 95.3% (1,267/1,330) of cases were residents of Ouagadougou; data on residency was unavailable for 67 cases. Outside of Ouagadougou, the largest case number was observed in Nioko II (11 cases), a temporary settlement on the northern border of Ouagadougou which has a population of low socioeconomic status.

In 2017, a second outbreak occurred between 10^th^ August and 29^th^ December with a peak of 57 cases on the 17^th^ October. During this time period, 25.1% (1,882/7,498) of patients presenting to Schiphra hospital with fever tested positive for dengue. The beginning of the outbreak coincided with the end of the rainy season in Burkina Faso which runs from June to September (Figure 1A) [14]. During both outbreaks, the majority of cases detected at the CMA laboratory resided in the west/south-west of Ouagadougou (Figures 1B and 1C). In 2016, the greatest number of cases originated in sector 17 (227 cases) and sector 19 (152 cases) (Figure 1B). In 2017, the greatest number of cases originated in the south-west and central regions of the city, and the highest number of cases was again reported in sector 17 (449) (Figure 1C).

Blood samples from febrile patients in 2016 were also assessed for *P. falciparum* malaria; 5.9% of the patients (292/4,939) were positive for *P. falciparum*. Co-infection of dengue infection and malaria was identified in 1.4% (39/2,762) of cases; however, dengue infection and malaria were negatively associated (*p* < 0.01).

## 4 Discussion

Despite the samples from this study originating from a single laboratory, we identified a higher number of dengue cases than those estimated by the WHO and those identified during the outbreak in the health facility-based surveillance study [11]; hence, results presented here complement these data. Results shown managed to capture an acute outbreak that differs from the research study [11], which conducted fever-based community surveillance. The major differences are that only RDT results were used to confirm dengue cases and no convalescent sera and confirmatory testing by RT-PCR had been executed. When adding up the numbers from the WHO report, the Lim et al. [11] study and the results shown here, the number of cases officially reported seem to significantly underestimate the magnitude of the outbreaks. Lim and colleagues’ study further highlighted the importance of dengue RDTs usage in routine care to address the issue of under-diagnosis as well as underreporting in the surveillance system [11].

We observed that the peak number of cases followed the rainy season in 2016 and 2017; such a seasonal observation has been observed previously [15, 17]. The difference in reporting numbers is also in line with the fact that dengue fever is potentially not sufficiently recognized and reported in Africa due to a variety of reasons, i.e. concomitant diseases exhibiting similar symptoms, low awareness of clinicians and nurses and, importantly, the lack of systematic diagnostic testing. Given that the SD Bioline Dengue Duo RDT has shown varying levels of sensitivity and specificity [12, 13], the recently reported lower sensitivity of 46.8% in the Brazilian study [13] suggests that half of the cases actually remain undetected and the disease burden is potentially even higher. In the study conducted by Lim et al., RT-PCR was used as confirmatory testing and subtyping (i.e. not performed on a random sample subset, but mainly on likely positive samples) [11]. Therefore, the study was not designed to allow a comparison of performance of different dengue tests [11].

In addition, although the reference laboratory was centrally located, we observed a disproportionate number of dengue cases originating from the west/south-west region of Ouagadougou. This might be due to environmental factors such as increased number of water bodies or, as we postulate, due to the fact that the number of healthcare facilities in these areas is lower, and febrile patients preferably refer to the CMA Schiphra facilities.

Data presented by the WHO and Lim et al. demonstrated the circulation of three different dengue serotypes during the 2017 and 2016 outbreak, respectively; DENV-2 being the most predominant serotype [10, 11]. Antibodies against one distinct serotype can induce an immunological response against an alternative serotype, but may not neutralize the virus, which may lead to a phenomenon known as antibody-dependent enhancement (ADE). ADE may occur following one or multiple dengue infections, in particular when infected a second time, resulting in more severe disease manifestations including dengue hemorrhagic fever and dengue shock syndrome (severe dengue). More evidence is needed, particularly on the occurrence of severe dengue, but the simultaneous circulation of three different dengue serotypes may potentially be one explanation of the relatively high case fatality rate observed in the recent outbreaks in Burkina Faso. ADE complicates the development of dengue vaccines, requiring that relevant antibodies against all serotypes be present and persist. The recently proposed label change for the recommended target population for Dengvaxia® underscores that risk, as vaccination of naïve individuals, during longer-term follow-up, may be associated with increased susceptibility to severe dengue following a subsequent dengue virus infection [16]. Given the potential impact of large dengue outbreaks on healthcare systems, it may be important to consider dengue vaccines as part of an integrated program of dengue control in Burkina Faso.

Dengue fever, unlike *P. falciparum* malaria, is observed principally in densely populated areas. *Aedes aegypti* prefers to breed in receptacles that collect water including car tires, ponds and water storage jars. Mosquitos of the *Anopheles* genus associated with transmitting malaria, are not typically found in urban settlements, which generally lead to a lower risk of malaria in cities in comparison to more rural locations. Furthermore, the blood feeding behaviour of the two mosquitos differs substantially. *Anopheles* species feeds from dusk till dawn, while *A. aegypti* predominantly feeds during daylight [17]. The characteristics of these vectors are consistent with the negative association observed between malaria and dengue.

The study has limitations. First, we only obtained cases from a single laboratory in Ouagadougou, therefore, we are unable to account for cases reported at other healthcare facilities. This limitation may account for the higher number of cases in the west/south-western areas of the city as the number of healthcare facilities is potentially lower than in the more central parts of the city. Therefore, case numbers may have been biased by referral patterns or differences in healthcare-seeking behaviour. However, there are very few laboratories in the city and the availability of a low-cost RDT for dengue at CMA resulted in >65% of patients being referred from across the city. Second, RDT results were not further confirmed by anti-DENV IgM capture ELISA or PCR, which may result in a number of false positives, leading to elevated case numbers. However, such confirmatory testing was performed in the Lim et. al study [11].

## 5 Conclusions

Improved surveillance and the routine use of an RDT resulted in the detection of outbreaks with increased dengue case numbers in Burkina Faso. These cases were detected in addition to a study conducted by another group at the same time, indicating that the burden of dengue fever is potentially higher than assumed and that clinical case definition for dengue is insufficient for dengue diagnosis. The comparison of results from several sources shows that adequate surveillance and epidemiological studies are paramount to enable the scientific community to create a situational awareness for this disease, which is severely under-recognized in Africa. Therefore, more resources should be invested into dengue surveillance in sub-Saharan Africa to ensure that high risk areas and high-risk populations are identified to support vaccine introduction rapidly upon the availability of such knowledge.

## Declarations

### Author contribution statement

J. Im, R. Balasubramanian and F. Marks: Conceived and designed the experiments; Analyzed and interpreted the data; Wrote the paper.

M. Ouedraogo, L. R. Wandji Nana and A. B. Soura: Performed the experiments; Contributed reagents, materials, analysis tools or data.

T. van Pomeren: Performed the experiments.

K. Prifti, H. J. Jeon, S. Baker, J. H. Kim, J. D. Clemens, C. G. Meyer, A. Haselbeck and J. K. Lim: Analyzed and interpreted the data; Wrote the paper.

O. D. Mogeni: Analyzed and interpreted the data.

### Funding statement

F. Marks, H. J. Jeon, A. Haselbeck, O. D. Mogeni, J. Im, T. van Pomeren were supported by a grant from the 10.13039/100000865Bill & Melinda Gates Foundation (OPP1127988).

### Competing interest statement

The authors declare no conflict of interest.

### Additional information

No additional information is available for this paper.
